# Critical Fluctuations in Cortical Models Near Instability

**DOI:** 10.3389/fphys.2012.00331

**Published:** 2012-08-20

**Authors:** Matthew J. Aburn, C. A. Holmes, James A. Roberts, Tjeerd W. Boonstra, Michael Breakspear

**Affiliations:** ^1^School of Mathematics and Physics, The University of QueenslandBrisbane, QLD, Australia; ^2^Systems Neuroscience Group, Queensland Institute of Medical ResearchBrisbane, QLD, Australia; ^3^The Black Dog Institute and School of Psychiatry, University of New South WalesSydney, NSW, Australia; ^4^Research Institute MOVE, VU University AmsterdamAmsterdam, Netherlands; ^5^The Royal Brisbane and Women’s HospitalBrisbane, QLD, Australia

**Keywords:** neural mass model, Hopf bifurcation, critical fluctuations, autocorrelation

## Abstract

Computational studies often proceed from the premise that cortical dynamics operate in a linearly stable domain, where fluctuations dissipate quickly and show only short memory. Studies of human electroencephalography (EEG), however, have shown significant autocorrelation at time lags on the scale of minutes, indicating the need to consider regimes where non-linearities influence the dynamics. Statistical properties such as increased autocorrelation length, increased variance, power law scaling, and bistable switching have been suggested as generic indicators of the approach to bifurcation in non-linear dynamical systems. We study temporal fluctuations in a widely-employed computational model (the Jansen–Rit model) of cortical activity, examining the statistical signatures that accompany bifurcations. Approaching supercritical Hopf bifurcations through tuning of the background excitatory input, we find a dramatic increase in the autocorrelation length that depends sensitively on the direction in phase space of the input fluctuations and hence on which neuronal subpopulation is stochastically perturbed. Similar dependence on the input direction is found in the distribution of fluctuation size and duration, which show power law scaling that extends over four orders of magnitude at the Hopf bifurcation. We conjecture that the alignment in phase space between the input noise vector and the center manifold of the Hopf bifurcation is directly linked to these changes. These results are consistent with the possibility of statistical indicators of linear instability being detectable in real EEG time series. However, even in a simple cortical model, we find that these indicators may not necessarily be visible even when bifurcations are present because their expression can depend sensitively on the neuronal pathway of incoming fluctuations.

## Introduction

Computational models of neocortex and other brain structures have proved very useful for a range of research problems in neuroscience (Braun and Mattia, 2010; Friston and Dolan, 2010). Interpreting empirical data using dynamical models is particularly fruitful in neuroimaging, where underlying processes are obscured by the low temporal resolution of fMRI or the coarse spatial source resolution of electroencephalography (EEG)/MEG. This allows testing of hypotheses about internal dynamical mechanisms (e.g., Freyer et al., 2012) and, through model inversion, the estimation of neural and connectivity parameters that cannot be observed directly (Friston et al., 2003). In contrast to modeling at the microscopic scale, where the range of dynamics of healthy neurons is known to include non-linear behavior such as limit cycles, modeling at the larger scale of mesoscopic neural masses, or neural fields often assumes that the dynamics at this scale operate close to a stable fixed point where input fluctuations result in only small and brief perturbations of the population state. This premise is predicated on the *diffusion approximation* that states that correlations amongst neuronal inputs are reduced as the size of the population increases (for review, see Deco et al., 2008). This approach enables the calculation of spectra from the composition of transfer functions, a powerful technique that allows physiological parameters to be estimated from non-invasive functional neuroimaging (Friston et al., 2003) and neurophysiological (van Albada et al., 2010) data.

Dynamic instabilities in models at the larger scale of neural masses have typically been associated with the pathological activity of epileptic seizures (Wendling et al., 2000; Robinson et al., 2002; Breakspear et al., 2006). However, empirical data shows that such instabilities may also underlie healthy neural activity (Freyer et al., 2009, 2011). Indeed, the Jansen–Rit neural mass model (Jansen and Rit, 1995) and its derivatives (Wendling et al., 2002; David and Friston, 2003; Zavaglia et al., 2006; Moran et al., 2007; Sotero et al., 2007; Spiegler et al., 2010) reach bifurcations where fixed points become linearly unstable while still within the healthy physiological range of parameters. In fact, oscillations in the model output that have been identified with normal cortical alpha activity have been shown to arise from limit cycle activity following a supercritical Hopf bifurcation (Grimbert and Faugeras, 2006; Spiegler et al., 2010).

The term “linear instability” here does not necessarily imply that the dynamics of the system as a whole lose stability. Indeed, in the case of the supercritical Hopf bifurcation, stability of the attractor is maintained as it deforms continuously from a stable fixed point to a stable limit cycle, which then increases in size in the phase space. Hence, there is no discontinuous transition. The distinction is that the dominant dynamics in the system are no longer linear. The presence of quadratic and higher order flow terms that become significant in the neighborhood of a bifurcating fixed point have a profound influence on the system’s statistical properties and its response to stochastic perturbations.

The putative presence of linear instabilities in healthy, mesoscopic cortical activity is ultimately an empirical question that must be answered with reference to the theory of non-linear stochastic dynamical systems. For a wide range of systems, statistical measures such as increased autocorrelation, increased variance, and bistable switching have been proposed as generic indicators that the system is losing linear stability on approaching a bifurcation (Scheffer et al., 2009; Kelso, 2010). Increased autocorrelation length is a direct consequence of critical slowing down, which occurs as the strength of attraction to a stable fixed point becomes weaker before changing to equally weak repulsion. Long-range correlations may also reveal a transition from exponential to power law relaxation in the vicinity of linear instabilities as a result of the higher order (non-linear) flow terms.

Within neuroscience, statistical indicators of bifurcations have been studied at a range of scales, in both computational models and empirical analyses. In the context of single neuron models, increase of variance close to a bifurcation and the spectral peak near a Hopf bifurcation have been examined (Steyn-Ross et al., 2006). Spectral features and variance close to instability have been explored in large-scale mean field corticothalamic models (Robinson et al., 1997, 2002; Roberts and Robinson, 2012) and mean field models of the brainstem and hypothalamus (Robinson et al., 2010). Slowing down, instability, and bifurcations have also been studied at the highest level of brain function, particularly in human movement. For example, increased variance and critical slowing have been observed in human bimanual motor control (Kelso et al., 1986; Scholz et al., 1987) and are explained by a simplified phenomenological model of coordination (Haken et al., 1985).

In addition to the analyses of empirical data contained within these computational studies, signatures of transitions in neuroimaging data have been the subject of a number of predominantly empirical studies. Amplitude fluctuations of human brain oscillations have been shown to have long time autocorrelations with power law decay in EEG (Linkenkaer-Hansen et al., 2001), consistent with effects expected near linear instability. Scale-free cortical activity has also been reported in surface electrocorticogram (ECoG) activity, although the significance, scaling coefficient, and likely mechanisms remain contested (Bedard et al., 2006; Miller et al., 2009; He et al., 2010). Similarly, Stam and de Bruin (2004) reported scale-free fluctuations in the degree of synchronization between surface EEG recordings. These findings are consistent with prior reports of intermittent non-linear structure within (Stam et al., 1999) and between (Breakspear and Terry, 2002) surface EEG channels. More recently, Freyer et al. (2009) found that 10 Hz oscillations showed intermittent switching between two distinct bistable modes, although the dwell times within each mode followed a stretched exponential, not a power law decay.

The objectives of the present study are to examine linear instabilities in the Jansen–Rit model, a closed set of equations describing the activity of a small cortical region and one of the simplest cortical neural mass models. At the same time it is a base upon which many extensions and derivative models have been built (Wendling et al., 2002; David and Friston, 2003; Zavaglia et al., 2006; Moran et al., 2007; Sotero et al., 2007; Spiegler et al., 2010). The phenomena which we report in this simple model therefore highlight the possibility of similar behavior in a wider class of models. We focus on one key indicator of linear instability (autocorrelation length) and one important bifurcation (supercritical Hopf). Time series for each neural population in the model are generated for sets of parameters approaching a bifurcation. We then test whether the autocorrelation indicator of proximity to bifurcation is reliably detectable in the time series of the pyramidal population and also examine scaling properties of fluctuations in this time series. In this way we explore whether simple bifurcations at the population scale have the potential to contribute to indicators such as lengthened autocorrelation times and power law scaling of fluctuations reported in human EEG data.

## Materials and Methods

### Jansen–Rit neural mass model

**Figure 1:**
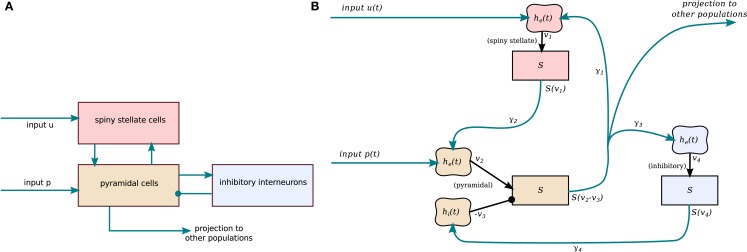
**Schematic connectivity of the Jansen–Rit model**. **(A)** Basic connectivity diagram showing the three neuronal populations, their excitatory (arrows) and inhibitory (circle) connections, and inputs from outside the local cortical region (*u* and *p*). **(B)** A block diagram then summarizes how this translates directly to a mathematical model: linear filter boxes labeled *h*_e_(*t*) and *h*_i_(*t*) model the mean response of excitatory and inhibitory synapse populations respectively, including their postsynaptic dendritic filtering. Sigmoid boxes (denoted *S*) represent conversion of mean summed soma membrane potential to mean output firing rate. Connectivity constants γ_1_ to γ_4_ model the number and strength of connections between populations.

Building on the earlier work of Lopes da Silva et al. (1974) and Wilson and Cowan (1972), Jansen and Rit developed a simple computational model of a small cortical region (Jansen et al., 1993; Jansen and Rit, 1995). The model produces an output signal similar to spontaneous EEG alpha oscillations, and also shows responses similar to evoked potentials following pulsatile input. The Jansen–Rit model is a closed set of differential equations that describe the local average states of three interconnected neural populations (Figure 1), excitatory interneurons, pyramidal cells, and inhibitory interneurons. Here we follow David and Friston (2003) in identifying the excitatory interneurons in the model with layer IV spiny stellate cells. The spiny stellate and pyramidal neurons are both excitatory and both populations receive external input, although only the pyramidal cells project out of the local region.

Each second order equation in the model corresponds to a population of synapses and their postsynaptic dendritic processes (Freeman, 1992; Deco et al., 2008). Critically damped second order linear filters describe the time course of the population mean of postsynaptic potentials, further dispersed due to variability of parameters within each population. This mean behavior summarizes both synaptic and dendritic dynamics of the individual neurons. For excitatory and inhibitory synapses respectively these filters are expressed by the differential operators

(1) $$L_{\text{e}}=\frac{1}{H_{\text{e}}\kappa_{\text{e}}}\left(\frac{d^{2}}{dt^{2}}+2\kappa_{\text{e}}\frac{d}{dt}+\kappa_{\text{e}}^{2}\right),$$

(2) $$L_{\text{i}}=\frac{1}{H_{\text{i}}\kappa_{\text{i}}}\left(\frac{d^{2}}{dt^{2}}+2\kappa_{\text{i}}\frac{d}{dt}+\kappa_{\text{i}}^{2}\right),$$

where the scalar parameters *H*_e_ and *H*_i_ determine the maximum amplitude of the postsynaptic population response to excitatory and inhibitory inputs, respectively. The rate constants κ_e_ and κ_i_ determine the time scale of these population responses. As the synaptic filters are linear, synapses with different source or target populations can be merged where synapses are assumed to have the same aggregate properties. For example Jansen and Rit (1995) consolidated their original model to just three second order equations, as it was implicitly assumed that excitatory and inhibitory interneuron populations would always have identical states up to a scaling constant. Following synaptodendritic filtering, fluctuations in membrane potential sum in the cell soma and lead to changes in the average population firing rate. The sigmoid function

(3) $$S\left(v\right)=\frac{2e_{0}}{1+\exp\left[\rho_{1}\left(\rho_{2}-v\right)\right]},$$

describes how the mean firing rate of a neural population depends on the mean soma membrane potential *v*, incorporating the dispersion of responses due to variability in the parameters and underlying neuronal states (Marreiros et al., 2008). Parameters *e*_0_, ρ_2_, and ρ_1_ determine the maximum firing rate, threshold potential, and sensitivity, respectively.

We express the Jansen–Rit model as a set of four second order differential equations, thus allowing both pyramidal and spiny stellate populations separately to receive extrinsic input. We follow the variable and parameter names of Moran et al. (2007). The dynamical variables *v*_1_, *v*_2_, and *v*_4_ represent the positive contributions to population mean soma potentials by excitatory synapses targeting spiny stellate, pyramidal, and inhibitory interneuron populations, respectively. Variable *v*_3_ represents the negative contribution to the mean soma potential of the pyramidal population originating from inhibitory synapses. Thus the resulting mean soma potential of the pyramidal population is *v*_2_ − *v*_3_. This is taken as the main output of the model (Jansen and Rit, 1995; David et al., 2005) because the size and orientation of the apical dendrites of pyramidal neurons mean that pyramidal activity is most closely associated with EEG signals. These equations are given by

$$\begin{array}{llll} L_{e}v_{1} & = & \gamma_{1}S\left(v_{2}-v_{3}\right)+\left\langle u\right\rangle +\sigma_{u}\xi_{u}\left(t\right), & \text{(4)}\\ L_{e}v_{2} & = & \gamma_{2}S\left(v_{1}\right)+\left\langle p\right\rangle +\sigma_{p}\xi_{p}\left(t\right), & \text{(5)}\\ L_{i}v_{3} & = & \gamma_{4}S\left(v_{4}\right), & \text{(6)}\\ L_{e}v_{4} & = & \gamma_{3}S\left(v_{2}-v_{3}\right). & \text{(7)} \end{array}$$

Equation 4 describes excitatory synaptic input targeting the spiny stellate population. Eqs 5 and 6 describe excitatory and inhibitory synaptic input to the pyramidal population, respectively. Equation 7 describes excitatory synaptic input to the inhibitory interneuron population. Parameters 〈*u*〉 and 〈*p*〉 are the mean per neuron external input firing rates to the cortical region, targeting spiny stellate, and pyramidal populations, respectively. Langevin white noise terms ξ_*u*_(*t*) and ξ_*p*_(*t*) in the extrinsic input represent the fluctuations in the input firing rates, with σ_*u*_ and σ_*p*_ denoting their standard deviations. Scalar connectivity constants γ_1_ to γ_4_ represent at the population scale the number and strength of connections between the three neural populations.

This system of equations is equivalent to a single eight-dimensional stochastic first order differential system:

(8) dv=f(v)dt+G dW(t),

where matrix elements of *G* determine the cross-correlation of noise inputs to the pyramidal and spiny stellate populations. This is the equation that we integrate numerically.

Table 1 lists the values of parameters used for all simulations in this study; they are the standard parameter values introduced by Jansen and Rit (1995).

**Table 1:** **Jansen–Rit standard parameter values**.

Parameter	Value	Description
*H*_e_	3.25 mV	Maximum amplitude of the excitatory postsynaptic population response
*H*_i_	22.0 mV	Maximum amplitude of the inhibitory postsynaptic population response
κ_e_	100 s^−1^	Rate constant for postsynaptic population response to excitatory input
κ_i_	50 s^−1^	Rate constant for postsynaptic population response to inhibitory input
*e*_0_	2.5 s^−1^	Half of the maximum population mean firing rate
ρ_2_	6.0 mV	Population mean firing threshold potential
ρ_1_	0.56 mV^−1^	Firing rate sigmoid function voltage sensitivity parameter
γ_1_	135	Connectivity constant: pyramidal to spiny stellate
γ_2_	108	Connectivity constant: spiny stellate to pyramidal
γ_3_	33.75	Connectivity constant: pyramidal to inhibitory interneurons
γ_4_	33.75	Connectivity constant: inhibitory interneurons to pyramidal

Jansen and Rit (1995) themselves focused on numerical simulations of this non-linear model. Through a survey of the simulated behavior with physiologically realistic parameters, they observed a variety of noise-driven rhythmic behaviors consistent with human alpha and beta rhythms. Wendling et al. (2000) studied the emergence of “spike-wave” oscillations resembling epileptic activity when the ratio of excitation to inhibition was increased. Bifurcations in this model where subsequently examined by Grimbert and Faugeras (2006) who treated the input *p* as the bifurcation parameter in order to understand better the original simulation results of Jansen and Rit (limit cycle beyond a Hopf bifurcation causing alpha oscillations) and Wendling et al. (the emergence of a large amplitude non-harmonic oscillator near a sniper bifurcation). More recently, Spiegler et al. (2010) performed a more general bifurcation analysis that included time scale parameters and analyzed the presence of qualitatively different oscillatory regimes.

### Bifurcation parameters

In the original model (Jansen et al., 1993), both pyramidal and excitatory interneuron populations were the targets of extrinsic inputs, with the two inputs being always proportional (fully correlated). In the model of Jansen and Rit (1995), all extrinsic input was delivered to the pyramidal neurons only, with external stimulation of the other population dropped.

David and Friston (2003) revisited the Jansen–Rit model, in particular explicitly identifying the “excitatory interneuron” population of the original model with spiny stellate cells in layer IV of the neocortex. Their motivation was to send extrinsic input to the layer IV spiny stellate cells in the model rather than to the pyramidal cells. This was arguably a more realistic model of connectivity for input representing thalamocortical sensory afferents. However the equations as published retained the pyramidal-only input of the original Jansen–Rit model.

Moran et al. (2007), in the context of Dynamic Causal Modeling (DCM, a framework for model selection and parameter estimation), extended the Jansen–Rit model with several innovations, including firing rate adaptation, recurrent inhibition, and a differently shaped sigmoid function. In particular Moran et al. did change the target of the extrinsic input to be the spiny stellate population, as foreshadowed by David and Friston (2003). We refer to this model as the Moran–Friston model hereafter.

For the present study we minimally extend the Jansen–Rit model, so that extrinsic input can be delivered either to the pyramidal population (as in Jansen and Rit, 1995), the spiny stellate excitatory population (as in Moran et al., 2007) or more realistically a combination of the two. In this way, the system input can be varied continuously from the Jansen–Rit design to the Moran–Friston design or anywhere in between. In addition, for the case of input to both populations, these two inputs can be chosen as uncorrelated, fully correlated, or partially correlated in their fluctuations. Hence we study the bifurcations of this model as input is varied in the combined (*u*, *p*) plane. This subset of parameter space includes a one-dimensional space explored by Jansen and Rit containing a supercritical Hopf bifurcation studied by Grimbert and Faugeras (2006) that is within the physiological range of parameters. We present the bifurcation analysis in Section “Bifurcation diagram.”

### Numerical simulation and analysis

The model is a system of stochastic differential equations (SDEs) with additive noise. Equation 8 is integrated numerically using the Heun algorithm, which is applicable to SDEs in Stratonovich form (Rümelin, 1982). This ensures that noise amplitude is scaled in appropriate proportion to the square root of the integration time step. We use an integration time step of 0.2 ms. The first transient 5 s of each simulation is discarded from further analysis.

As reviewed in the introduction, the approach to linear instabilities in systems of equations such as Eqs 4–7 is widely assumed to cause changes in the autocorrelation length and/or a peak in the power spectral density function (in the case of a Hopf bifurcation). This is because it is often assumed that the linear treatment of these systems – which predicts both an enhancement of spectral peaks and a lengthening of the autocorrelation time – can be extrapolated from the setting when the system is linearly stable to when it is in the neighborhood of a bifurcation.

To estimate the normalized autocorrelation function of the resulting time series we first normalize each time series to a mean of 0 and standard deviation of 1, and then compute the cross-correlation of the series with itself applying unbiased normalization,

(9) $${\hat{R}}_{\text{xx,unbiased}}\left(m\right)=\frac{1}{N-m}\overset{N-m-1}{\underset{n=0}{\sum }}x_{n+m}x_{\text{n}}\left(m\geq 0\right),$$

where *m* is the lag expressed as number of samples (Orfanidis, 1996). In each case we compute autocorrelation at lag times from 0 to 1/4 of the total time series length for further analysis. Since time series are generated in the vicinity of Hopf bifurcations with natural frequency about 11 Hz, the autocorrelation functions all have a strong 11 Hz component. Because we are primarily interested in the decay of the amplitude of this autocorrelation over a longer time scale, each simulation is repeated 16 times with identical parameters to generate 16 sample paths each of duration 600 or 1,800 s. The autocorrelation function is calculated as described above for each sample path separately. The decay is then quantified by calculating the modulus of the Hilbert transform of the normalized autocorrelation functions computed above. The pointwise mean and standard deviation of this autocorrelation amplitude across 16 sample paths are then plotted. Power spectra are estimated using the Welch algorithm with Hamming window and a segment length of 80,000 samples or 16 s.

The full MATLAB code implementing the model, integration, and time series analyses is available from the authors on request.

## Results

### Bifurcation diagram

From the earlier bifurcation analysis of Grimbert and Faugeras (2006) the model is known to have a supercritical Hopf bifurcation when the pyramidal input *p* = 89.8 s^−1^ and the other parameters are set to the values used by Jansen and Rit (1995). This assumed no input to the excitatory (spiny stellate) population. We label this bifurcation point H1; it has mean input firing rate 〈*p*〉 = 89.8 s^−1^ to the pyramidal population and zero input to the spiny stellate population (i.e., 〈*u*〉 = 0, σ_*u*_ = 0). This maps directly back to the original Jansen–Rit model with pyramidal-only input. Matching the effective noise level used by Jansen and Rit (correcting a scaling error in the original paper) is achieved by allowing *p* to fluctuate with standard deviation σ_*p*_ = 0.5390 s^−1^.

To examine the difference between cases where input is provided in different ratios to the spiny stellate population and pyramidal population, we continue the bifurcation point H1 in the (*u*, *p*) plane in parameter space, using the numerical continuation package MATCONT (Dhooge et al., 2003).

**Figure 2:**
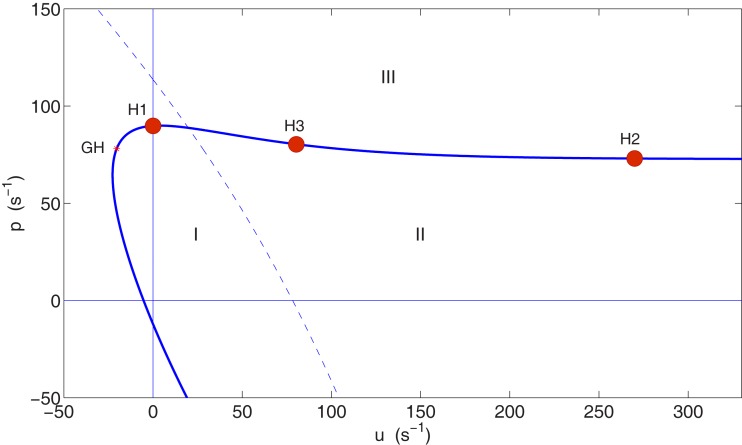
**Bifurcation diagram in the (*u*, *p*) parameter plane showing Hopf curve (thick, solid curve) and the location of the chosen Hopf bifurcation points H1, H2, and H3 on this curve**. A generalized Hopf bifurcation (GH) marks the transition from subcritical Hopf points (the curve below GH) to supercritical Hopf points (the curve continuing beyond GH). Regions where *p* < 0 or *u* < 0 are non-physical. Below the Hopf curve (regions I and II) a stable fixed point exists, which gradually loses linear stability as the curve is approached. Above the Hopf curve (region III) this point has lost linear stability and become a stable limit cycle. The dashed line is a curve of fold bifurcation points. In region II a single stable fixed point exists. In region I the system is bistable with a second stable fixed point also existing, at lower excitation.

Figure 2 shows the bifurcation diagram in the (*u*, *p*) plane. This plane is a two-dimensional slice through the larger parameter space of the model, so that a curve in this plane corresponds to a surface in parameter space. The Hopf curve is almost horizontal for *u* > 0, implying that the level of pyramidal cell stimulation required to reach the supercritical Hopf bifurcation in the model (*p* ∼ 75–90 s^−1^) is roughly independent of the level of spiny stellate cell stimulation for *u* ≳ 0. For comparison with H1, we select point H2 on this same surface of supercritical Hopf points, but this time with greater mean input to the spiny stellate population (〈*u*〉 = 270 s^−1^) than to the pyramidal population (〈*p*〉 = 73 s^−1^). The magnitude of fluctuations $\sqrt{\sigma_{u}^{2}+\sigma_{p}^{2}}$ in the input is kept the same as at H1, with a standard deviation of σ_*u*_ = 0.5203 s^−1^ in the spiny stellate input and σ_*p*_ = 0.1407 s^−1^ in the pyramidal input.

### Autocorrelation indicator behaves differently at H1 and H2

For each of the bifurcation points we simulate the dynamics at four locations in parameter space: the approach to the bifurcation from the linearly stable side (two points), at the bifurcation point (one point), and beyond the bifurcation (one point). In each case the output pyramidal time series (*v*_2_ − *v*_3_) is the focus of our analysis.

Each simulation is performed separately with parameter values fixed at these different values, rather than performing a single dynamic simulation with sliding parameters. This approach allows the time series analyzed at a fixed parameter value to be approximately stationary (provided the total time simulated is long enough) so that statistics for the process at that parameter point can be estimated from a finite time series. Where variance is considered as an indicator of instability this approach also avoids any spurious short-time increases in variance due to the dynamically shifting range of the system in phase space, as distinct from increased noise-driven variance at the new parameter values (Kuehn, 2011).

**Figure 3:**
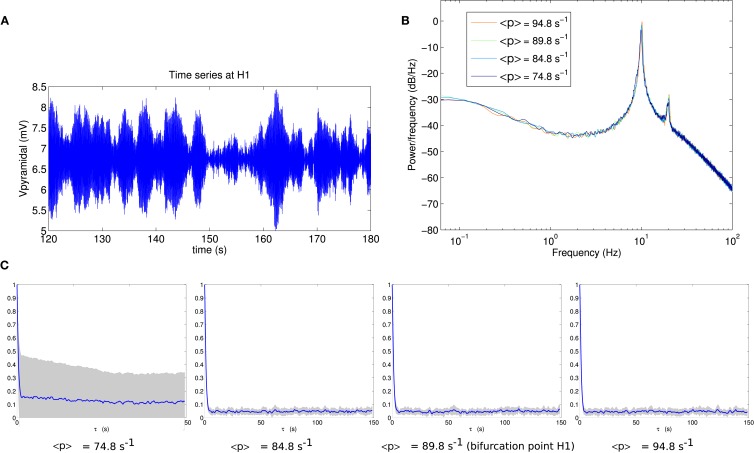
**(A)** Example section of simulated time series at the bifurcation point H1. **(B)** Power spectrum at H1. **(C)** Autocorrelation amplitude at points in parameter space approaching the bifurcation point H1 (<*p*> = 74.8 s^−1^, <*p*> = 84.8 s^−1^), at the bifurcation point H1 (<*p*> = 89.8 s^−1^) and beyond the bifurcation point (<*p*> = 94.8 s^−1^). The line indicates the mean over 16 trials and the gray area indicates one standard deviation.

To determine the effect of proximity to a bifurcation on the fluctuation statistics, we analyze the approach and passage through bifurcations H1 and H2. Figure 3 shows the results for bifurcation H1. An exemplar pyramidal time series (Figure 3A) reveals a fluctuating oscillatory system, whose power spectrum (Figure 3B) peaks at the frequency of the Hopf instability, namely 11 Hz. The series of panels in Figure 3C shows that when approaching and passing point H1 (from left-to-right), the autocorrelation time stays approximately constant.

**Figure 4:**
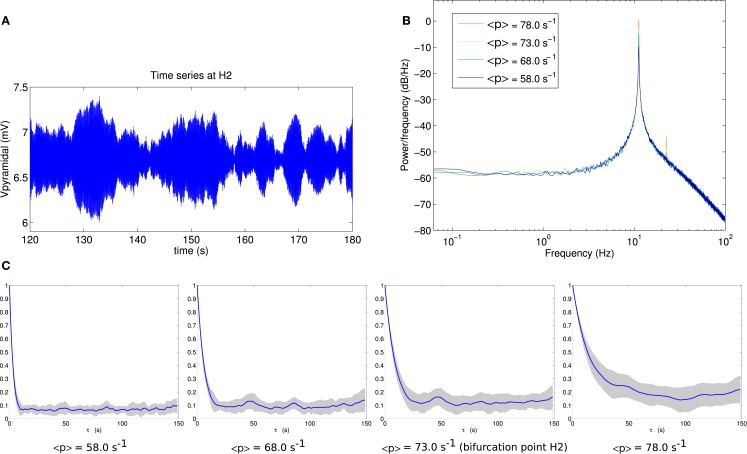
**(A)** Example section of simulated time series at the bifurcation point H2. **(B)** Power spectrum at H2. **(C)** Autocorrelation amplitude at points in parameter space approaching the bifurcation point H2 (<*p*> = 58.0 s^−1^, <*p*> = 68.0 s^−1^), at the bifurcation point H2 (<*p*> = 73.0 s^−1^) and beyond the bifurcation point (<*p*> = 78.0 s^−1^). The line indicates the mean over 16 trials and the gray area indicates one standard deviation.

For comparison, the corresponding analyses for bifurcation H2 are shown in Figure 4. By eye, the fluctuation envelope of the amplitude appears smoother. As is evident in Figure 4C, the autocorrelation amplitude decays much more slowly as the system approaches the bifurcation. At the bifurcation point H2 the autocorrelation remains above 20% of its zero-lag value at a lag of 15 s.

The variance of the output pyramidal time series increases as the bifurcation H1 is approached, with standard deviations of 0.4550, 0.5344, 0.5630, and 0.6110 mV at the four parameter points respectively. Approaching H2 this also occurs, with standard deviations of 0.1454, 0.2160, 0.2582, and 0.3449 mV respectively for the output time series. It is notable that in the vicinity of point H2, the standard deviation of the simulated pyramidal output time series is between 1.8 and 3.1 times smaller than in the vicinity of point H1, while autocorrelation times are roughly 7 times longer than at H1.

As expected for a Hopf bifurcation in a stochastic system the dynamics change gradually and continuously through the bifurcation (Rowat and Greenwood, 2011). The amplitude of oscillations increases when moving toward and beyond the bifurcation point as revealed by increased variance of the output time series. Close to the bifurcation point this reflects weakening of the stability of the (fixed point) attractor while beyond the bifurcation point it reflects increasing size of the (limit cycle) attractor. The increase in power at 11 Hz is visible in the power spectrum (Figure 3B).

The comparison of Figures 3C and 4C shows that autocorrelation is a useful and clearly visible indicator of linear instability in the vicinity of point H2, but not for point H1. This is despite these being points on the same surface of bifurcations with the same variance of input fluctuations.

We conjecture that the key difference between H1 and H2 is the orientation of the input fluctuations in phase space with respect to the two-dimensional center manifold of the bifurcation, which determines the specific directions in which linear stability is weakening. When close to the equilibrium point, the center manifold surface can be approximated by the center eigenspace of the bifurcation. Since the eigenvectors of the linearized system are far from orthogonal, the relevant reference plane to determine the noise component projected into the center eigenspace is the plane that is perpendicular to the stable eigenspace. For H1 the resulting projection of the noise vector onto this reference plane is cosα = 0.0031. For H2 the projection is cosα = 0.0008; i.e., the noise input has a projection onto that plane that is four times larger in the case of H1 than in the case of H2.

However, the comparison between points H1 and H2 does not by itself give strong support for this hypothesis, because there are several other factors that are significantly different between H1 and H2. In particular H2 has 3.1 times the total input firing rate of H1, so that on this basis the difference in autocorrelation could simply be due to greater level of excitation for point H2. This motivates the comparison constructed below.

### Autocorrelation depends on orientation of input fluctuations

**Figure 5:**
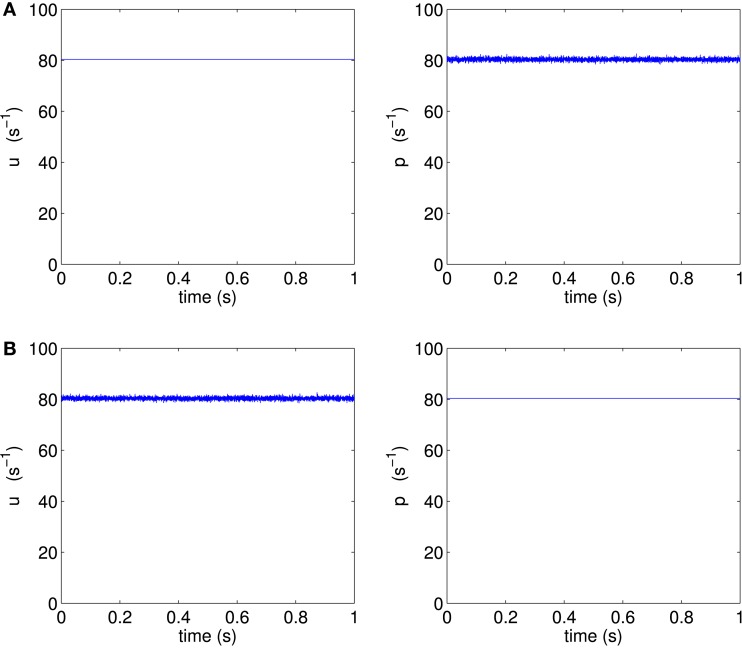
**One second of sample external inputs *u* and *p*, (A) for scenario H3p and (B) for scenario H3u**.

In order to separate the effect of different mean firing rates from the effect of different noise orientation, we construct two new scenarios H3p and H3u, where the only difference between them is the noise orientation; all other parameters are kept identical. We choose point H3 on the same bifurcation line of supercritical Hopf points, but with equal mean input firing rates to pyramidal and spiny stellate populations (mean input firing rate per neuron of 〈*u*〉 = 〈*p*〉 = 80.35 s^−1^). We simulate two scenarios at point H3 to test the conjecture, with both scenarios using the same values for all model parameters, and in particular with both scenarios using the same mean input firing rates, as illustrated in Figure 5.

We define the scenario H3p as the case where only the pyramidal input is allowed to fluctuate about its mean, while spiny stellate input is held steady at its mean value, corresponding to the parameters 〈*p*〉 = 80.35 s^−1^, σ_*p*_ = 0.5390 s^−1^, 〈*u*〉 = 80.35 s^−1^, and σ_*u*_ = 0 s^−1^.

Scenario H3u is defined as the case where only the spiny stellate input is allowed to fluctuate, while pyramidal input is held steady, corresponding to the parameters 〈*p*〉 = 80.35 s^−1^, σ_*p*_ = 0 s^−1^, 〈*u*〉 = 80.35 s^−1^, and σ_*u*_ = 0.5390 s^−1^.

By using these two constructed scenarios, all parameters in the simulation are kept identical between scenarios H3p and H3u except for the direction of the fluctuations of input in phase space, which is rotated in phase space from the pyramidal direction to the spiny stellate direction. Rotating the vector of fluctuations independently from the vector of mean inputs is non-physiological. The simulated results of the non-physiological scenarios H3p and H3u are used to shed light on the reason for different autocorrelation in the original realistic scenarios H1 and H2.

**Figure 6:**
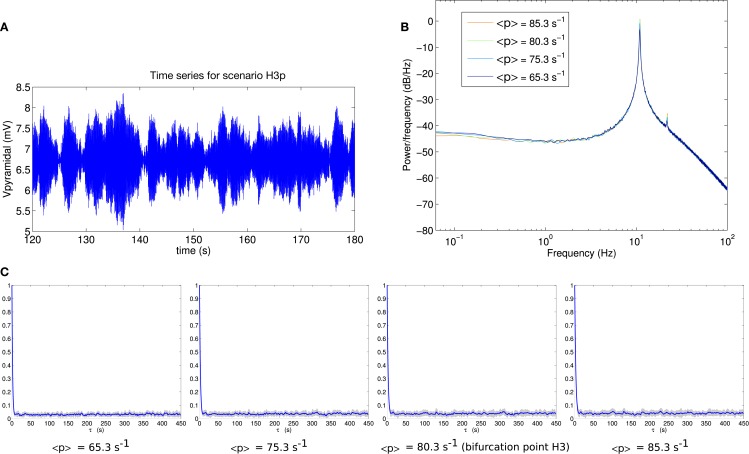
**(A)** Example section of simulated time series for scenario H3p. **(B)** Power spectrum at H3. **(C)** Autocorrelation amplitude at points in parameter space approaching the bifurcation point H3 (<*p*> = 65.3 s^−1^, <*p*> = 75.3 s^−1^) at the bifurcation point H3 (<*p*> = 80.3 s^−1^) and beyond the bifurcation point (<*p*> = 85.3 s^−1^). The line indicates the mean over 16 trials and the gray area indicates one standard deviation.

**Figure 7:**
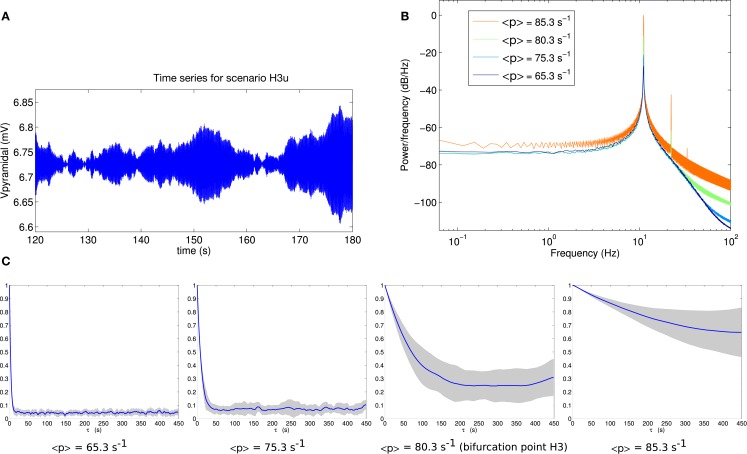
**(A)** Example section of simulated time series for scenario H3u. Note the *y*-axis scale is much smaller than that of Figure 6A, reflecting much smaller output variance in this case. **(B)** Power spectrum at H3. **(C)** Autocorrelation amplitude at points in parameter space approaching the bifurcation point H3 (<*p*> = 65.3 s^−1^, <*p*> = 75.3 s^−1^), at the bifurcation point H3 (<*p*> = 80.3 s^−1^) and beyond the bifurcation point (<*p*> = 85.3 s^−1^). The line indicates the mean over 16 trials and the gray area indicates one standard deviation.

Comparable analyses of these two scenarios are presented in Figures 6 and 7. The contrast between scenarios H3p and H3u is clear in Figures 6C and 7C. When the fluctuations are in the input to the pyramidal population (scenario H3p), the decay of autocorrelation amplitude changes little as the bifurcation point is approached. By contrast, when the fluctuations are in the input to the spiny stellate population (scenario H3u) the indicator of increased autocorrelation length is very prominent. A large increase in autocorrelation heralds the transition to linear instability in scenario H3u with significant autocorrelation at lags of up to 450 s. This indicator is much less evident in scenario H3p.

**Figure 8:**
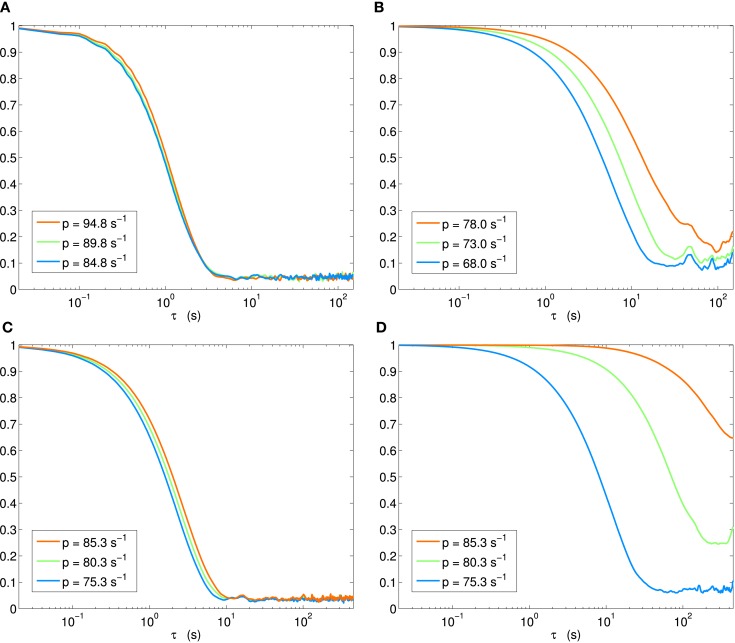
**Autocorrelation amplitude in log(delay)-linear(correlation) coordinates**. Each panel shows step immediately before (blue), at (green), and beyond (orange) Hopf bifurcation. **(A)** Scenario H1, **(B)** H2, **(C)** H3p, **(D)** H3u.

It is also instructive to view the autocorrelation amplitude with log scaling of the delay axes. The results for the four scenarios (H1, H2, H3p, H3u) we have thus far considered are shown in Figure 8. Whereas the autocorrelation length stays almost invariant across the bifurcation in scenario H1 (Figure 8A), a clear increase is seen in scenario H2 (Figure 8B). Where scenario H3p shows a small, but systematic lengthening (Figure 8C), a progression through the same points in parameter space – but now with input fluctuations aligned with the stable eigenspace – can again be seen to lead to a dramatic increase (Figure 8D).

In both scenarios H3p and H3u the variance of the output pyramidal time series increases as the bifurcation point H3 is approached, with standard deviations of 0.4884, 0.4730, 0.5134, and 0.5339 mV for H3p and 0.0188, 0.0295, 0.0873, and 0.3033 mV for H3u. The standard deviation is starkly different between these two scenarios, with standard deviation between 2 and 22 times smaller in scenario H3u than in scenario H3p. Thus changing the noise input direction results in both reduced variance and increased autocorrelation length. Variance also increases more rapidly in scenario H3u than H3p as the bifurcation is approached.

Relating this to the orientation of input noise, the contrast in alignment is even greater between scenarios H3p and H3u than in the comparison of H1 and H2. For H3p the projection of the input noise onto the reference plane perpendicular to the stable eigenspace is cosα = 0.003, i.e., noise input has a non-negligible component perpendicular to the stable eigenspace of the bifurcation near the equilibrium point, whereas for H3u the projection is cosα = 0.00006, i.e., the projection of the noise input onto the reference plane is 50 times smaller in the case of H3u than H3p.

### Input correlation and output variable not important

The results presented above are calculated from the pyramidal time series. Applying the same process to time series for the other two populations in the model (spiny stellate and inhibitory) shows that in each case, the results for autocorrelation decay and variance show the same behavior as the pyramidal time series. This is important, as it rules out the possibility that the autocorrelation difference results from different amounts of filtering between the noise input and the measured output.

In the case of point H2, both pyramidal input and spiny stellate input have fluctuations. To check whether correlations between the two input fluctuations are important to the results we examine the two extreme cases of independent and perfectly correlated inputs. Cross-correlation of input fluctuations does not affect the results: autocorrelation amplitude of the pyramidal output decays over a similar time scale whether the inputs to the two populations are independent or perfectly correlated.

### Scaling properties of output fluctuations

As reviewed earlier, long-tailed fluctuation distributions have been observed in the amplitude fluctuations of alpha (Freyer et al., 2009) and beta oscillations in scalp EEG data (Linkenkaer-Hansen et al., 2004). Therefore we study the statistical properties of fluctuations at and near the bifurcation points in scenarios H3u and H3p. In particular, we characterize fluctuations by the distributions of sizes and durations of excursions in the amplitude envelope of the detrended pyramidal time series. More specifically, we analyze the squared Hilbert amplitude, which is a measure of instantaneous power. We extract excursions above a threshold (sometimes termed “avalanches” in the literature) where each excursion is delineated by the time points at which the instantaneous power crosses the threshold from below and the next crossing from above. Fluctuation duration is thus the length of the time interval for which the power is above threshold, and we define fluctuation size to be the time integral of the instantaneous power over this interval (i.e., the area under the curve, a measure of energy in the fluctuation). We choose the threshold for each time series such that it approximately maximizes the number of identified fluctuations and falls in a regime where the fluctuation statistics are relatively insensitive to small changes in this value.

We analyze the fluctuation size and duration distributions following the methods of Clauset et al. (2009). For each set of fluctuation statistics we calculate the inverse cumulative distribution function and fit candidate distributions to the tail using the method of maximum likelihood: power law (the Pareto distribution), power law with exponential cutoff, lognormal, and exponential. Here the tail is all the data above a lower bound that we identify as the value that minimizes the Kolmogorov–Smirnov goodness-of-fit statistic between the power law model and the data (Clauset et al., 2009). This method of determining the range of the fit from the data strikes a balance between fitting too wide a range (i.e., outside the power law regime) and too narrow a range (i.e., throwing away data unnecessarily). We use the same fitting range for all four candidate distributions. We estimate a *p*-value for the fitted power law by comparing the data to 1,000 synthetic data sets drawn from a true power law, which accounts for whether the deviation between the data and the fitted power law is within the range expected for finite sampling of a true power law. The *p*-value is taken to be the fraction of synthetic data sets that deviate from the power law by at least as much as the data, and *p* > 0.1 indicates plausibility of the power law hypothesis (Clauset et al., 2009). We compare the fitted power law with alternative distributions using likelihood ratio tests. Significant deviation of the likelihood ratio from zero is tested using Vuong’s methods (Vuong, 1989). For the nested hypothesis of power law versus power law with cutoff (the latter family includes the former), the null hypothesis is that the power law is best-fitting distribution. For all other tests, the null hypothesis is that both distributions are equally far from the true distribution.

**Figure 9:**
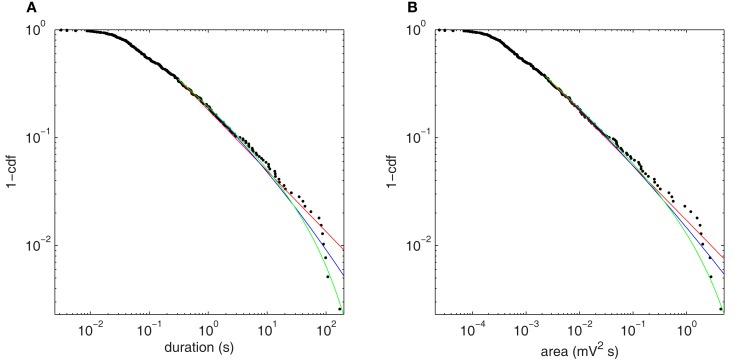
**Upper cumulative distributions of fluctuation statistics at the bifurcation point H3u, using squared Hilbert amplitude thresholded at 0.008 mV^2^, with power law (red), power law with exponential cutoff (green), and lognormal (blue) fits plotted for the fitted range of the tail**. **(A)** Fluctuation duration. **(B)** Fluctuation size as given by area under the curve.

Figure 9 shows the fluctuation distributions for H3u. The empirical distributions for both duration (Figure 9A) and area (Figure 9B) exhibit a scaling regime over approximately four orders of magnitude. The power law fits for duration and area have exponents 1.56 and 1.51, and *p*-values *p* = 0.27 and *p* = 0.72, respectively, and are thus consistent with the hypothesis that the true distribution is a power law. The fitted exponents depend weakly on the threshold value but the main finding of a broad scaling regime is unchanged. The lognormal and power law with exponential cutoff are *also* consistent with the data: the likelihood ratio tests do not distinguish between the lognormal and power law fits (duration: *p* = 0.15; area: *p* = 0.26), but favor the power law with exponential cutoff over both power law (duration: *p* = 0.016; area: *p* = 0.047) and lognormal (duration: *p* = 0.003; area: *p* = 0.004). The pure exponential distribution is strongly ruled out in all cases (*p* ≪ 0.001) and so is not shown.

**Figure 10:**
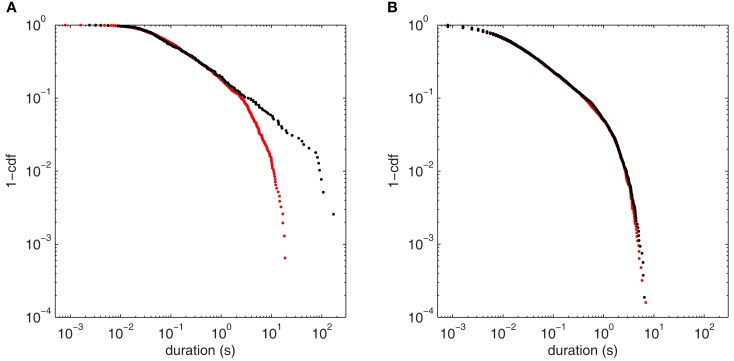
**Comparison of fluctuation duration distributions between points approaching (*p* = 65.3 s^−1^) and at the bifurcation point (*p* = 80.3 s^−1^) for the two noise input directions**. **(A)** Point H3u (black, threshold = 0.008 mV^2^) and nearby more stable point (red, threshold = 0.0002 mV^2^). **(B)** Point H3p (black, threshold = 0.4 mV^2^) and nearby more stable point (red, threshold = 0.18 mV^2^).

Approach to this bifurcation, shown in Figure 10, reiterates the autocorrelation results of Section “Autocorrelation Depends on Orientation of Input Fluctuations.” Near H3u (Figure 10A), the long scaling regime of Figure 9A (black) is significantly diminished away from the bifurcation (red), with few fluctuations having durations >10 s. Here, the pure power law is ruled out (*p* < 0.001), and the power law with exponential cutoff is strongly favored over all the alternatives tested. For comparison, Figure 9B shows fluctuations at the same bifurcation when noise enters almost perpendicular to the center eigenspace (scenario H3p). At the bifurcation (black), there is no clear scaling regime, and the distribution is essentially unchanged by moving to a more stable point in parameter space (red). The power law fit is ruled out for both points (*p* < 0.001), and again the power law with cutoff is strongly favored. Thus, as in the autocorrelation cases (Figures 7C and 8C), the fluctuation statistics clearly herald the approach to the bifurcation for H3u but only negligibly for H3p.

## Discussion

The relevance of these results to physiology is twofold. Firstly, we have demonstrated a fundamental limitation in the use of autocorrelation as an indicator of the loss of linear stability, a limitation which will apply when attempting to detect bifurcations from actual human EEG, EMG, and MEG time series. Secondly, the demonstration of both long autocorrelation times and scale-free temporal fluctuations in a simple, low-dimensional stochastic model informs the debate about whether the brain exhibits self-organized criticality, because it shows that these features can also arise from mechanisms other than a multi-scale critical phase transition.

Close to the supercritical Hopf bifurcation in the Jansen–Rit model, we have shown that when lengthened autocorrelation times and scale-free fluctuations manifest in any one cell population as indicators of approach to the bifurcation, then they are indeed detectable in the pyramidal time series that is most closely associated with EEG signals. The standard parameters of the model provide sufficiently large coupling between the three neural populations that lengthened autocorrelation is evident in all three populations when it is present in any of them.

When considering long time autocorrelation and scale-free fluctuations that are present in human EEG time series this suggests that in addition to the possibility that these could arise in the brain at the point of phase transition in a complex, multi-scale system (Linkenkaer-Hansen et al., 2004; Stam and de Bruin, 2004), there may also be a role for low-dimensional stochastic dynamics at the population scale in generating these indicators.

More importantly, we have shown that even in a very simplistic cortical model, these indicators can already be subtle in their dependency on neuronal inputs. Longer autocorrelation times are not guaranteed to be evident in the output just because there is a bifurcation where linear stability is lost. In particular we have shown that a change of the orientation in phase space of small fluctuations in the input can be sufficient to enhance or almost completely remove this indicator.

Jansen and Rit (1995) suggested that input to excitatory interneurons could be removed from the model, as input to the pyramidal population from coupled columns was expected to have the same effect. Our results show that when fluctuations in the input are taken into account, the statistical properties of the model output are sensitive to the choice of which neural population receives the extrinsic input.

The Jansen–Rit model is representative of a broad class of models that mathematically can be expressed as a composition of sigmoid functions and second order linear filters. It is worth noting that neural field models (such as Jirsa and Haken, 1996; Robinson et al., 1997), when restricted to spatially uniform solutions, can also be expressed in this mathematical form, with an additional critically damped second order linear filter capturing the time characteristics of local axonal propagation with a population spread of sources and axon parameters (Robinson et al., 1997). There are no particularities of the current model that suggest that the phenomena which we describe will be limited to this setting. The present results regarding fluctuation orientation hence speak broadly to the commonly employed neural mass and neural field models of large-scale neuronal activity.

### Opposite effect on autocorrelation and variance

Autocorrelation and variance of the output signal have been suggested as generic indicators of the approach to local bifurcation, as standard linear analysis shows they are both expected to increase as the bifurcation is approached and the real part of bifurcating eigenvalues approaches zero. We also observed that changing the orientation of input fluctuations can result in autocorrelation increasing at the same time as variance is decreased. Insight into these phenomena can be gained by considering the behavior of a simple low-dimensional linear stochastic system. In the one-dimensional linear case of an Ornstein–Uhlenbeck process, *dx* = −*axdt* + *bdW*, the (normalized) autocorrelation is given by exp(−*a*τ) and variance by 1/2 *b*^2^/*a*, so both increase as the size of the eigenvalue *a* approaches zero. In particular, variance increases linearly with the variance of noise input *b*^2^ (Gardiner, 2010). The same is true for a linearized two-dimensional system near a Hopf bifurcation (Steyn-Ross et al., 2006). If we naively assume that aligning input noise with the center eigenspace increases the amount of noise affecting the slow dynamical system of the center manifold, we would expect variance to be greater when the angle with the center eigenspace plane was smaller, which was not the case. From consideration of the normal form transformation (Roberts, 2008) it is rather the plane perpendicular to the stable eigenspace that should be relevant in determining the magnitude of noise driving the slow dynamics. Because the eigenvectors of the linearized Jansen–Rit system are far from orthogonal, that reference plane is almost normal to the center eigenspace plane, resulting in the observed reversal of the expected relationship between noise orientation and variance.

It may be possible to study these bifurcation indicators more specifically in a normal form model by considering a full center manifold reduction. Close to the bifurcation non-linear terms can result in multiplicative noise in the slow dynamical system of the center manifold (Roberts, 2008). These occur in addition to the simpler additive noise that results directly from linear transformation of the input noise terms but so far we have yet to calculate the magnitude or importance of these multiplicative noise terms in the present system. Furthermore, any local analysis of the behavior close to the equilibrium point is valid only for the case of small noise, so that the state of the system remains local to the equilibrium point. That is not necessarily the case for this system, as suggested by larger output standard deviation near the bifurcation seen in the cases of H1 and H3p, which is comparable to the amplitude of the subsequent limit cycles. This implies that the system is exploring a wider region of phase space compared to the cases with high autocorrelation (H2 and H3u). Thus the structure of flow in the phase space further from the equilibrium point may be directly responsible for the quickly decaying autocorrelation in those cases. In particular if the center manifold curves away from the center eigenspace, then at a sufficient distance from the equilibrium point the directions of noise input which are “well aligned” and “poorly aligned” with the manifold may be reversed.

### Power law scaling of output fluctuations

Analyzing the distributions of fluctuation sizes and durations, we observe the presence of a long power law scaling regime that extends over four orders of magnitude with a rapid truncation at the far right hand tail at the bifurcation when input fluctuations are normal to the reference plane. This power law scaling is not observed when input fluctuations have a significant projection onto the reference plane. Further away from the bifurcation, the power law regime extends for less than one order of magnitude so that the lengthy power law tail provides a signature of proximity to the bifurcation in that scenario.

A range of simple dynamical mechanisms are known to permit production of scale-free fluctuation structure of this kind. A relaxation process with a fractional operator formally yields a power law (Pareto) probability distribution of fluctuation durations (Sokolov and Klafter, 2005). Multiplicative noise (which arises when reducing oscillation dynamics of the model to two dimensions) can also in specific cases result in power law probability distributions (Anteneodo and Riera, 2005). However, the cause of the power law scaling of the distributions of fluctuations in our system is not yet determined.

### Future work

This study considered autocorrelation in the output of a single Jansen–Rit model region, representing a small area of cortex of the order of 2–3 mm^2^. For the question of potential detectability in EEG it remains to examine the effect on autocorrelation of combining the output of a large number of cortical regions, whose oscillations may be synchronized to a greater or lesser degree and where the output measurement function relating EEG to the combination of sources plays an important role.

Within the Jansen–Rit model we also observed indicators close to other bifurcation types, including switching between attractors in a bistable region near a cusp bifurcation and “flickering” or intermittent switching away from a stable fixed point in a monostable region near a sniper bifurcation, which are not explored further in this paper. Therefore it remains to examine the sensitivity of these and other indicators, such as mean switching times as bifurcations are approached, to noise orientation.

It is hoped that normal form analyses near the bifurcation will shed some light on the mechanism by which the input noise affects autocorrelation. A first step will be to examine a simpler normal form system displaying the same behavior, where exact control over the shape of the center manifold can be afforded, initially targeting the limiting case of small fluctuations. Such an analysis will serve to separate the generic local effects of the Hopf bifurcation from global behavior due to excursion of the state further from the equilibrium point.

Examination of a normal form system will also be key to determining the reason for the power law scaling of fluctuation statistics. The results presented in this paper show that some of the indicators of instability reported in human EEG also arise in the output of a simple neural mass model near linear instability.

While similar indicators can also emerge from a critical phase transition in a complex, multi-scale system, we have shown in the present study that some of the same indicators can arise in a very different way, from the low-dimensional stochastic dynamics at a single scale: the mesoscopic scale of interacting populations. As the field advances, it will become increasingly important to move away from a single umbrella notion of “criticality” in brain dynamics toward defining a number of exact, and possibly distinct, mechanisms responsible for correlations and scale-free fluctuations in the time and/or spatial domains. It is certainly possible at this stage that multiple mechanisms play a role.

## Conflict of Interest Statement

The authors declare that the research was conducted in the absence of any commercial or financial relationships that could be construed as a potential conflict of interest.

## Figures and Tables

**Figure A1:**
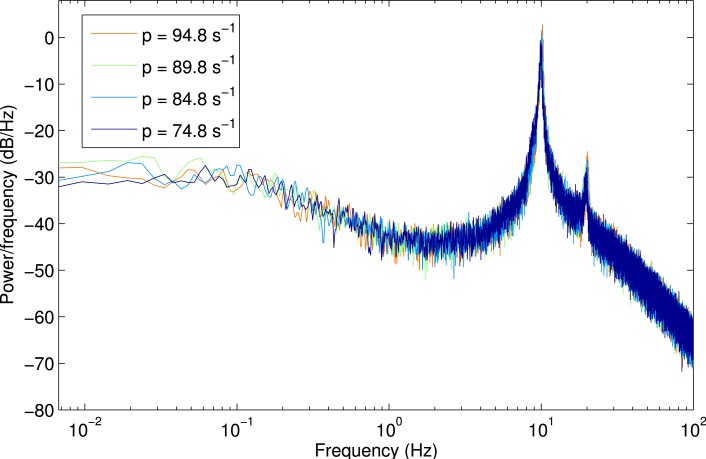
**Power spectrum at H1, using a larger window size of 150 s (750,000 samples) to show lower frequencies from 6.7 × 10^−3^ Hz**.

**Figure A2:**
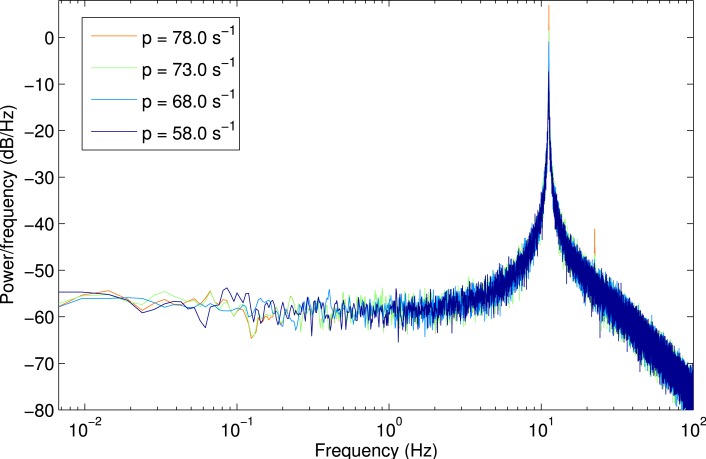
**Power spectrum at H2, using a larger window size of 150 s (750,000 samples) to show lower frequencies from 6.7 × 10^−3^ Hz**.

**Figure A3:**
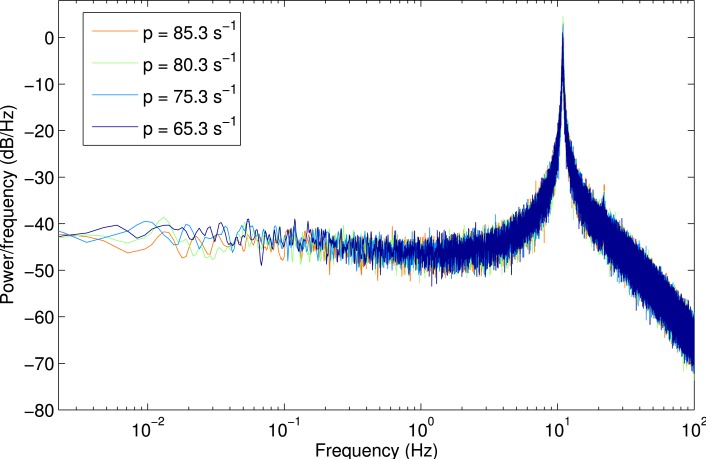
**Power spectrum for scenario H3p, using a larger window size of 450 s (2,250,000 samples) to show lower frequencies from 2.2 × 10^−3^ Hz**.

**Figure A4:**
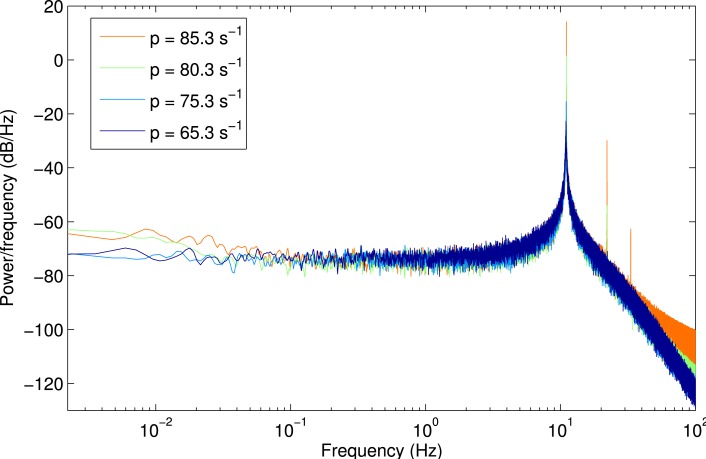
**Power spectrum for scenario H3u, using a larger window size of 450 s (2,250,000 samples) to show lower frequencies from 2.2 × 10^−3^ Hz**.
